# Determining the Time of Booster Dose Based on the Half-Life and Neutralization Titers against SARS-CoV-2 Variants of Concern in Fully Vaccinated Individuals

**DOI:** 10.1128/spectrum.04081-22

**Published:** 2023-07-10

**Authors:** Yu-Ching Dai, Yen-Chia Lin, Lauren L. Ching, Jih-Jin Tsai, Kyle Ishikawa, Wen-Yang Tsai, John J. Chen, Vivek R. Nerurkar, Wei-Kung Wang

**Affiliations:** a Department of Tropical Medicine, Medical Microbiology and Pharmacology, John A. Burns School of Medicine, University of Hawaii at Manoa, Honolulu, Hawaii, USA; b Pacific Center for Emerging Infectious Diseases, John A. Burns School of Medicine, University of Hawaii at Manoa, Honolulu, Hawaii, USA; c Department of Quantitative Health Sciences, John A. Burns School of Medicine, University of Hawaii at Manoa, Honolulu, Hawaii, USA; d Tropical Medicine Center, Kaohsiung Medical University Hospital, Kaohsiung, Taiwan; e Division of Infectious Diseases, Department of Internal Medicine, Kaohsiung Medical University Hospital, Kaohsiung, Taiwan; f School of Medicine, College of Medicine, Kaohsiung Medical University, Kaohsiung, Taiwan; Pontificia Universidad Católica de Chile

**Keywords:** severe acute respiratory syndrome coronavirus 2, variants of concern, neutralizing antibodies, half-life, mRNA vaccine

## Abstract

Although mRNA-based COVID-19 vaccines reduce the risk of severe disease, hospitalization and death, vaccine effectiveness (VE) against infection and disease from variants of concern (VOC) wanes over time. Neutralizing antibodies (NAb) are surrogates of protection and are enhanced by a booster dose, but their kinetics and durability remain understudied. Current recommendation of a booster dose does not consider the existing NAb in each individual. Here, we investigated 50% neutralization (NT_50_) titers against VOC among COVID-19-naive participants receiving the Moderna (*n* = 26) or Pfizer (*n* = 25) vaccine for up to 7 months following the second dose, and determined their half-lives. We found that the time it took for NT_50_ titers to decline to 24, equivalent to 50% inhibitory dilution of 10 international units/mL, was longer in the Moderna (325/324/235/274 days for the D614G/alpha/beta/delta variants) group than in the Pfizer (253/252/174/226 days) group, which may account for the slower decline in VE of the Moderna vaccine observed in real-world settings and supports our hypothesis that measuring the NT_50_ titers against VOC, together with information on NAb half-lives, can be used to dictate the time of booster vaccination. Our study provides a framework to determine the optimal time of a booster dose against VOC at the individual level. In response to future VOC with high morbidity and mortality, a quick evaluation of NAb half-lives using longitudinal serum samples from clinical trials or research programs of different primary-series vaccinations and/or one or two boosters could provide references for determining the time of booster in different individuals.

**IMPORTANCE** Despite improved understanding of the biology of SARS-CoV-2 (severe acute respiratory syndrome coronavirus 2), the evolutionary trajectory of the virus is uncertain, and the concern of future antigenically distinct variants remains. Current recommendations for a COVID-19 vaccine booster dose are primarily based on neutralization capacity, effectiveness against circulating variants of concern (VOC), and other host factors. We hypothesized that measuring neutralizing antibody titers against SARS-CoV-2 VOC together with half-life information can be used to dictate the time of booster vaccination. Through detailed analysis of neutralizing antibodies against VOC among COVID-19-naive vaccinees receiving either of two mRNA vaccines, we found that the time it took for 50% neutralization titers to decline to a reference level of protection was longer in the Moderna than in the Pfizer group, which supports our hypothesis. In response to future VOC with potentially high morbidity and mortality, our proof-of-concept study provides a framework to determine the optimal time of a booster dose at the individual level.

## INTRODUCTION

Unbridled transmission of severe acute respiratory syndrome coronavirus 2 (SARS-CoV-2) has resulted in the emergence of several variants of concern (VOC), including the alpha (B.1.1.7), beta (B.1.351), gamma (P1), delta (B.1.617.20), and omicron (B.1.1.529) variants (1–3). While COVID-19 vaccines are effective at reducing risk of severe disease, hospitalization, and death (4, 5), studies have shown reduction and waning of vaccine effectiveness (VE) against infection and disease caused by VOC and highlighted the need for in-depth studies of the durability of vaccine-induced immunity (6–15).

Because vaccine-induced neutralizing antibodies (NAb) against VOC, a well-characterized surrogate of protection in both human and nonhuman primate studies, reduce and wane over time (16–27), breakthrough infections have been documented after two or three doses of mRNA-based COVID-19 vaccines (28–32). Although recent studies have reported that a third dose (booster) of mRNA vaccine enhanced NAb against VOC and reversed waning VE (33–39), a better understanding of the kinetics and durability of NAb induced by the two mRNA vaccines and other vaccines is critically needed to determine the best timing of a booster vaccine (14, 15). With different waves of the pandemic, from the alpha and delta to omicron and its lineages under monitoring, the recommendation of a booster dose to reduce the morbidity and mortality caused by the next VOC has been a chasing and evolving process based on the latest neutralization capacities of vaccinee sera against the VOC and concurrent VE data, which can be delayed on many occasions, together with risk factors such as age, underlying diseases, and the immunocompromised conditions of different populations. Even within the healthy adult population, there are individual variations in vaccine-induced immunity, particularly existing NAb titers in different individuals, which were not taken into consideration in developing the current recommendation of a vaccine booster. We hypothesized that measurement of NAb titers against SARS-CoV-2 VOC together with information on the half-lives of NAb against VOC can be used to dictate the time of booster vaccination. In this study, we investigated NAb against different VOC among COVID-19-naive participants following two doses of Moderna or Pfizer vaccine and calculated the half-lives and time at which 50% neutralization (NT_50_) titers declined to a level equivalent to 50% inhibitory dilution (ID_50_) of 10 international unit (IU)/mL in each participant (40). This study provides a proof-of-concept approach to determine the optimal time of a booster vaccine against future VOC at the individual level.

## RESULTS

### Higher NT_50_ titers against VOC in COVID-19-recovered vaccinees than in naive vaccinees.

**FIG 1:**
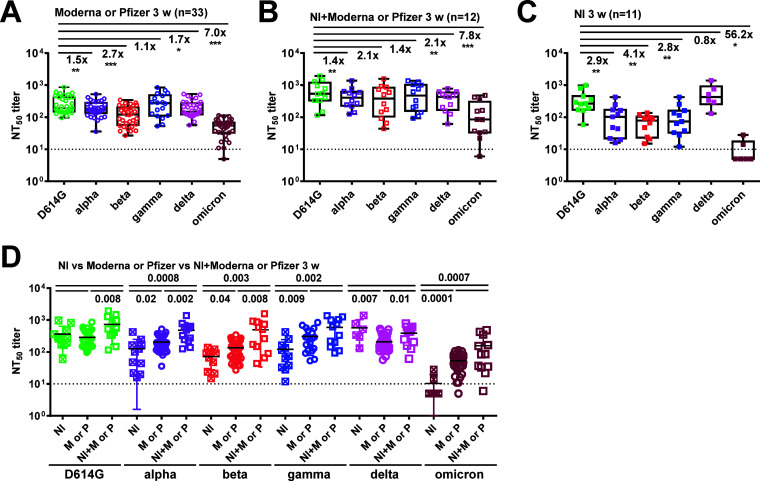
NT_50_ titers against SARS-CoV-2 D614G strain and VOC (alpha, beta, gamma, delta, and omicron) based on pseudovirus neutralization test among COVID-19 cases following natural infection (NI), COVID-19-naive, and COVID-19-recovered participants who had received Moderna or Pfizer vaccine. (A and B) COVID-19-naive (*n* = 33) (A) and COVID-19-recovered (*n* = 12) (B) participants at 3 weeks following Moderna or Pfizer vaccine. (C) COVID-19 cases at 3 weeks following NI (*n* = 11). (D) Comparison of NT_50_ titers against VOC between the three groups. Data are the means of duplicates from one experiment. Dotted lines: NT_50_ titer = 10. For box and whisker graphs in panels A to C: “number ×” = the median fold-reduction in NT_50_ titers against each VOC compared with that against the D614G strain. *, *P* < 0.05 and ≥0.01; **, *P* < 0.01 and ≥0.001; ***, *P* < 0.001; Wilcoxon signed-rank test. In panel D, numbers = *P* values, two-tailed Mann-Whitney test between two groups. VOC are color-coded: green, D614G; purple, alpha; red, beta; blue, gamma; pink, delta; brown, omicron.

To investigate whether previous COVID-19 exposure affected vaccine-induced NAb against the wild-type D614G strain and VOC, we first examined pseudovirus NT_50_ titers in plasma of COVID-19-naive (*n* = 33) compared with COVID-19-recovered (*n* = 12) participants at 3 weeks following two doses of Moderna or Pfizer vaccine and convalescent-phase plasma from COVID-19 cases with natural infection (NI) (*n* = 11) (41, 42). In agreement with previous reports, the NT_50_ titers against VOC decreased compared with those against the D614G strain in the COVID-19-naive vaccinees, with the highest fold of reduction to the omicron variant (7.0-fold reduction compared with D614G strain), followed by the beta, delta, alpha, and gamma variants (Fig. 1A) (43). A similar trend was observed in the COVID-19-recovered vaccinees and NI group (Fig. 1B and C), except that the latter had a higher fold reduction in NT_50_ titers against the omicron and less against the delta variant. Notably, the NT_50_ titers against 4 VOC (alpha, beta, gamma, and omicron) were significantly higher in the COVID-19-naive or COVID-19-recovered vaccinees compared with the NI group (Fig. 1D). The NT_50_ titers against the D614G and 3 VOC (alpha, beta, and delta) were significantly higher in the COVID-19-recovered than in the COVID-19-naive vaccinees (Fig. 1D).

### Comparable NT_50_ titers against VOC in COVID-19-naive individuals up to 7 months following two doses of Moderna or Pfizer vaccine.

**FIG 2:**
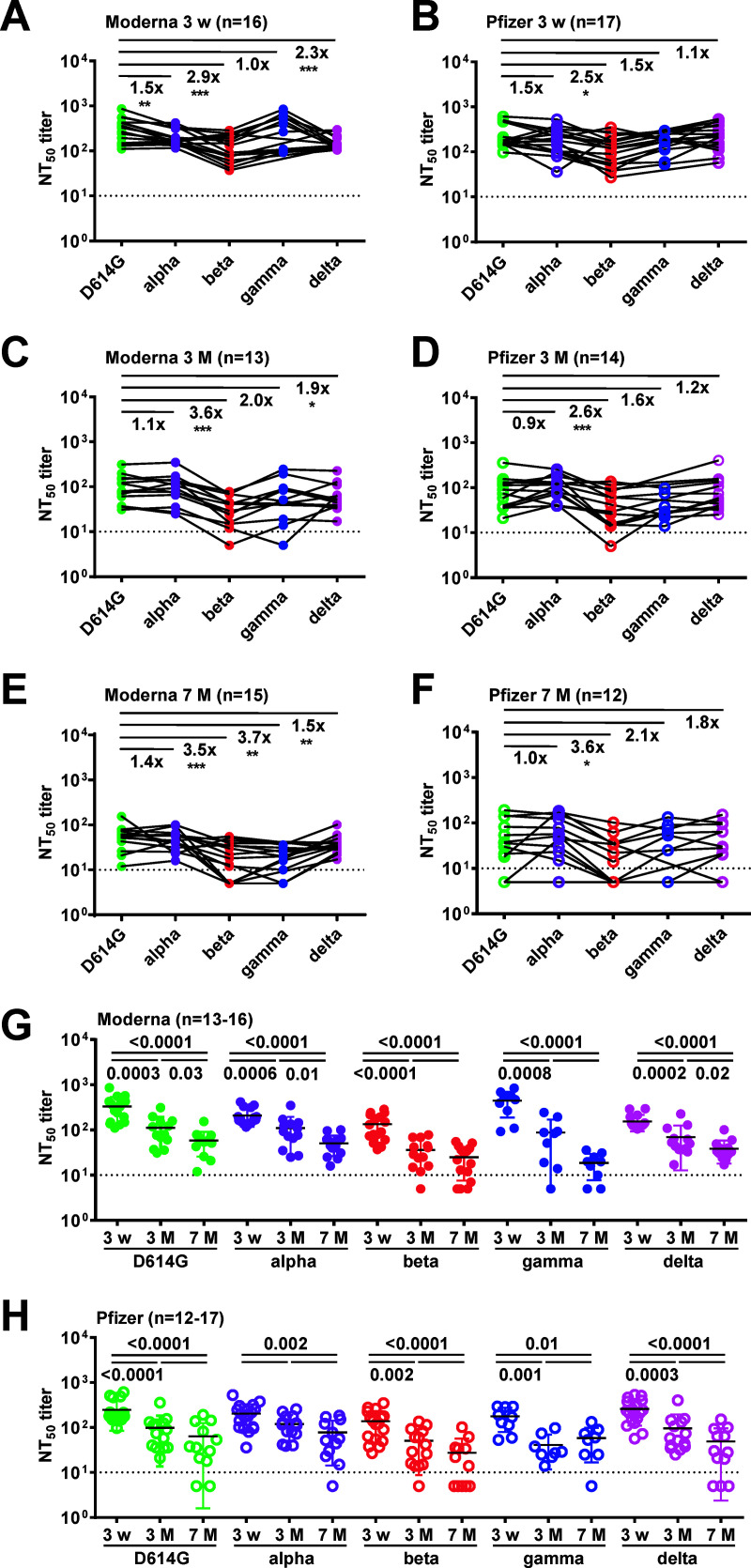
NT_50_ titers against SARS-CoV-2 D614G strain and VOC (alpha, beta, gamma, and delta) based on pseudovirus neutralization test among COVID-19-naive participants receiving Moderna or Pfizer vaccine. (A to F) NT_50_ titers at 3 weeks (A and B), 3 months (C and D) and 7 months (E and F) following two doses of mRNA vaccine. In panels A to F, “number ×” = mean fold-reduction in NT_50_ titers against each VOC compared with that against the D614G strain. *, *P* < 0.05 and ≥0.01; **, *P* < 0.01 and ≥0.001; ***, *P* < 0.001; Wilcoxon signed-rank test. (G and H) NT_50_ titers against the D614G and VOC declined over time within the Moderna (G) and Pfizer (H) groups. Numbers = *P* values, two-tailed Mann-Whitney test. Data are the means of duplicates from one experiment. Dotted lines: NT_50_ titer = 10. Color code for each VOC is the same as in Fig. 1.

We next compared the NT_50_ titers against VOC between COVID-19-naive participants who had received the Moderna (*n* = 26) or Pfizer (*n* = 25) vaccine over time (Table S1 in the supplemental material). Because the samples of study participants were collected between July 2020 and October 2021, prior to the emergence of the omicron variant, NT_50_ titers against the omicron variant over time were not included in subsequent analyses. Consistent with previous findings, the NT_50_ titers against VOC decreased compared with those against the D614G strain in both the Moderna and Pfizer groups, with the highest fold reduction against the beta variant, followed by the gamma, delta, and alpha variants (Fig. S2A and S2B). The NT_50_ titers at each time point revealed a similar pattern of reduction (Fig. 2A to F). When we compared the two mRNA vaccines at 3 weeks, the NT_50_ titers against the D614G strain, beta, and delta variants were comparable except for the gamma and delta variants (Fig. S2C). There was no difference in the NT_50_ titers against the D614G strain or VOC at 3 or 7 months except for the delta variant at 7 months (Fig. S2D and E). Similarly, there were no differences noted in sampling days, age, gender, or race/ethnicity (Table S1) except that the sampling days at the first time point (3 weeks) were earlier in the Pfizer than in the Moderna group (mean: 16.3 versus 19.3 days). Within the Moderna group, a significant decline in NT_50_ titers was observed for all VOC between 3 weeks and 3 or 7 months, and between 3 and 7 months for some variants (D614G, alpha, and delta variants); a similar trend of declining NT_50_ titers was observed between 3 weeks and 3 or 7 months within the Pfizer group except for the alpha variant (Fig. 2G and H).

### Lower rate of decline in NAb against VOC in the Moderna compared to the Pfizer group.

**FIG 3:**
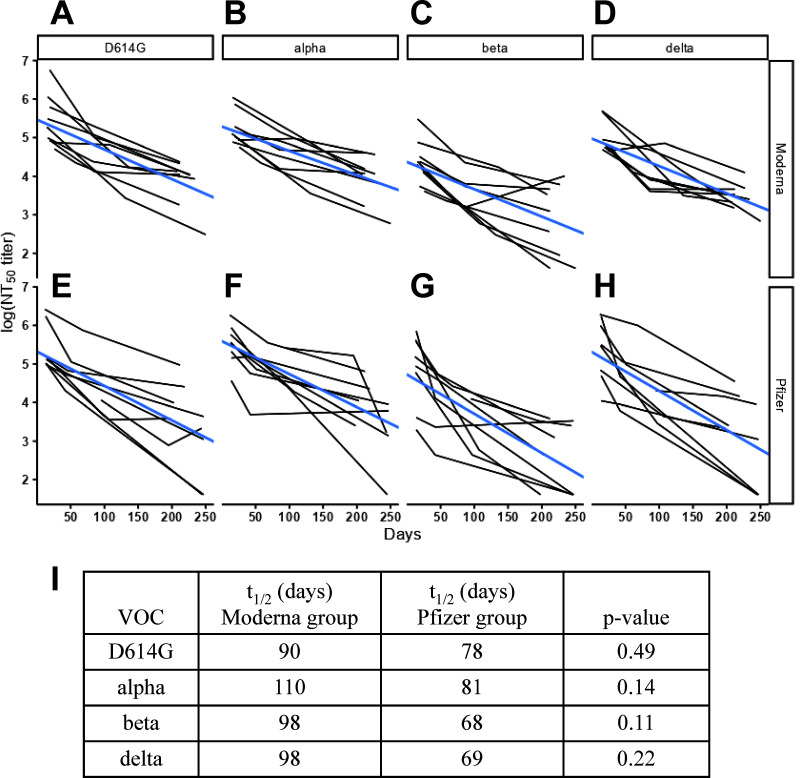
Decline of NT_50_ titers against VOC in the Moderna and Pfizer groups. For sequential samples of the Moderna (A to D) and Pfizer (E to H) groups (*n* = 27 from 9 participants in each group), NT_50_ titers were log-transformed and analyzed using an LME model to determine the half-life (t_1/2_) of NAb against the D614G strain (A and E) and each VOC including the alpha (B and F), beta (C and G), and delta (D and H) variants for each individual. (I) Summary of the half-life of NAb for the Moderna and Pfizer groups. Data are the means of duplicates from one experiment. The decline of NT_50_ titers against each VOC estimated by LME is shown in blue lines (*lme4* package of R version 4.1.2). *P* values were based on the Wilcoxon rank-sum tests comparing the Moderna and Pfizer groups.

To examine the decline in NAb over time, we plotted the NT_50_ titers against each VOC and sampling days following the second dose for the Moderna and Pfizer groups. For initial samples (*n* = 26 and *n* = 25 for the Moderna and Pfizer groups, respectively), we used a nonlinear regression method to determine the best fit curve and estimate the half-life (Fig. S3). The half-lives of NAb against the D614G strain, alpha, beta, gamma, and delta variants were longer for the Moderna group than for the Pfizer group (*P* = 0.04, two-tailed Mann-Whitney test). For sequential samples (*n* = 9 each group), we used the linear mixed effects (LME) regression model with a single slope to determine the half-life (Fig. 3 and Fig. S4). A trend of longer half-lives of NAb against the D614G strain, alpha, beta, and delta variants was observed in the Moderna group (90 to 110 days) compared to the Pfizer group (68 to 81 days) (*P* = 0.49, 0.14, 0.11, and 0.22, respectively, Wilcoxon rank-sum test).

**FIG 4:**
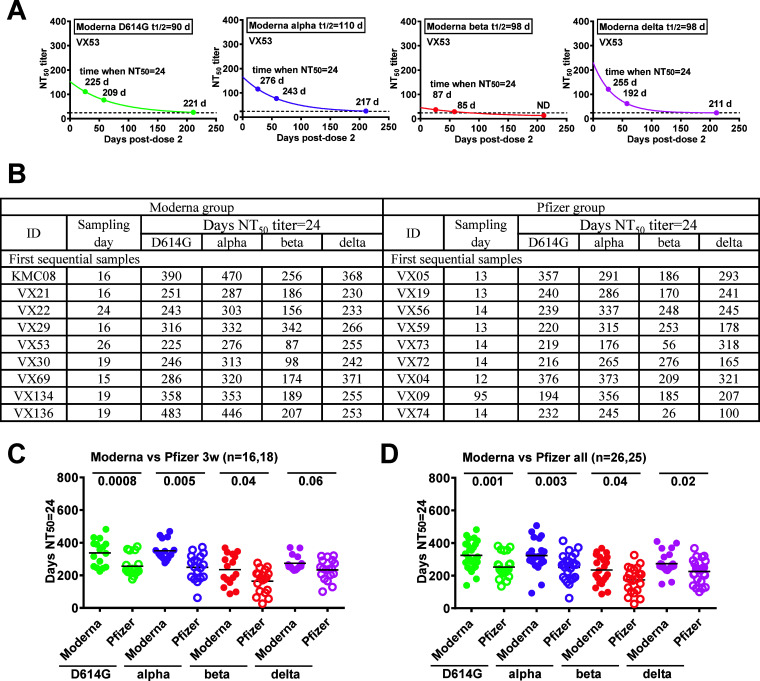
The time following the second dose of the Moderna or Pfizer vaccine it took NT_50_ titers against each VOC to decline to 24. (A and B) Based on the half-life and NT_50_ titer against each VOC on different sampling days, the time after the second dose required for the NT_50_ titer to decline to 24 was calculated for a Moderna vaccinee (VX53) (A) and for each participant in the Moderna and Pfizer groups (B). ND = not determined because NT_50_ titer < 24. Dashed lines, NT_50_ titer = 24. (C and D) Comparison of the time required for NT_50_ titers to decline to 24 for each VOC between the Moderna and Pfizer groups based on the 3-week samples (C) and all initial samples (D). Numbers = *P* values, two-tailed Mann-Whitney test. Color code for each VOC is the same as in Fig. 1.

A recent phase 3 trial of the Moderna vaccine reported that a 78% vaccine efficacy against symptomatic infection can be achieved by a ID_50_ of 10 IU/mL at 4 weeks after the second dose, which corresponds to NT_50_ titer of 24 by a calibration factor of 0.413 using a lentivirus-based pseudovirus neutralization test similar to ours (26, 40, 44). We first used the half-lives of NAb against each VOC and NT_50_ titers at different time points from one participant (VX53) to calculate the time at which NT_50_ titers declined to 24, demonstrating the feasibility of this approach (Fig. 4A). We then focused on the NT_50_ titers at the first time point (~3 weeks), which were close to the peak following the second dose of mRNA vaccine (14), to calculate the time for each participant in the Moderna and Pfizer groups (Fig. 4B, Table S2). For the D614G strain, this ranged from 225 to 483 or 176 to 376 days following the second dose of the Moderna or Pfizer vaccine, respectively; this is consistent with individual variation in NT_50_ titers within each group. A similar trend was observed for the alpha, beta, and delta variants (Table S2, Fig. 4C). To further evaluate the projected times at which NT_50_ titers reached 24, we examined some data (NT_50_ titers) from the third time point, which were within 30 days of the projected times based on the first time point, to see whether they were close to 24. Most of the NT_50_ titers were close to 24, consistent with the prediction; namely, they were higher or lower than 24 when the third time points were before or after the projected times, respectively (Fig. S5), supporting our calculation. In agreement with the lower rate of decline in NT_50_ titers in the Moderna than in the Pfizer group, the time it took for NT_50_ titers to decline to 24 was significantly longer in the Moderna group compared with the Pfizer group, except for the delta variant (Fig. 4C, *P* = 0.06). We further used NT_50_ titers at 3 and 7 months to calculate the time (Table S2), and found that, including all initial samples together, the average times for the D614G, alpha, beta, and delta variants in the Moderna group were 325, 324, 235 and 274 days, respectively, which were significantly longer than those in the Pfizer group (253, 252, 174 and 226 days, respectively) (Fig. 4D). These findings suggest that the average time for a booster dose for the D614G and alpha variants is 10.8 and 8.4 months, whereas it is 7.8 and 5.8 months for the beta variant, and 9.1 and 7.5 months for the delta variant, following the second dose of the Moderna and Pfizer vaccine, respectively.

## DISCUSSION

A recent study reported progressive waning of VE against SARS-CoV-2 severe disease (89% to 64% from 1 to 4 months) and infection of any severity with a lower rate of waning for the Moderna compared to the Pfizer group (VE: 71% versus 49% after 4 months) (45). A similar finding was reported by two other studies (46, 47). Although the larger mRNA dose (100 μg) in the Moderna vaccine has been suggested to account for the slower waning in VE, a comparative study of the kinetics and durability of NAb induced by the two mRNA vaccines is lacking (46, 47). Our findings of comparable NT_50_ titers against VOC up to 7 months following two doses of Moderna or Pfizer vaccine suggest that higher NAb titers (induced by the larger dose of mRNA in the Moderna vaccine) may not account for the slower waning in VE (46, 47). In agreement with this, comparable CD4 and CD8 T-cell recognition of VOC following the two vaccines was recently reported (48). We found that the longer half-lives of NAb induced by the Moderna vaccine and thus the lower rates of decay may account for the slower decline in VE of the Moderna vaccine observed in the real world. The half-lives of pseudovirus NAb determined in our study (68 to 110 days) were in the same range as previously reported for NI (90 days) or vaccination (63 to 140 days, average of two-phase decay) (15, 49). Although the mechanism underlying the longer half-life of NAb in the Moderna compared with the Pfizer group was not addressed in this study, several possibilities, such as differences in lipid nanoparticle components and dose intervals, may affect the responsiveness and durability of memory B or T-cells; these remain to be investigated (50).

Consistent with the report from the Moderna vaccine trial that vaccine efficacies of 78% and 91% against symptomatic infection can be achieved by pseudovirus ID_50_ of 10 and 100 IU/mL, respectively, a study of the AstraZeneca vaccine reported that an 80% vaccine efficacy of can be achieved by an ID_50_ of 26 IU/mL (25, 26). Based on the 10 IU/mL ID_50_ from the Moderna study as a reference (NT_50_ titer = 24 in our assay) (26, 44), the NAb half-life, and NT_50_ titers against each VOC at a given time, we calculated the ideal time of a booster dose for each participant. It is worth noting that this NT_50_ titer of 24 was based on the calibration factor of 0.413 from a similar pseudovirus system (26, 44). Because different assays may have different calibration factors (51), we performed the same calculation assuming a calibration factor of 1 and found a similar trend in that the time it took for NT_50_ titers to decline to 10, corresponding to an ID_50_ of 10 IU/mL and also the limit of detection of our neutralization test, was longer in the Moderna than in the Pfizer group (Fig. S6).

Although an extended dosing interval between the first and second dose of mRNA vaccine (primary series) has been reported to increase NAb titers, the time (booster interval) at which an optimal response to the booster, including higher and more durable NAb titers, might be obtained remains unknown (50, 52, 53). This fundamental question for booster strategy should be addressed by well-designed studies. However, with the continuing and widespread transmission of the omicron variant and its lineages in the community, reinfections and breakthrough infections are likely to occur frequently (54, 55). This scenario makes the study of COVID-19-naive or -recovered participants following primary series without breakthrough infection or reinfection to study the kinetics of pure vaccine-induced immunity and the time interval for an optimal booster response very challenging. The window period for such studies might have passed. In our study, all samples were tested by anti-nucleocapsid enzyme-linked immunosorbent assay (ELISA) to rule out breakthrough infections. Given the increasing herd immunity through vaccinations or infections together with the attenuated replication and pathogenicity of the omicron variant, most reinfections and breakthrough infections have resulted in asymptomatic infection or mild disease; however, the concern of new VOC with increased virulence remains, especially in the coming fall and winter seasons (56–60). Our study not only provided information about the kinetics of NAb against different VOC up to 7 months for COVID-19-naive participants receiving two doses of mRNA vaccines, but also a simple method to determine the optimal time of a booster dose at the individual level, which could facilitate the development of relevant protocols in the future and inform the change in practice and guidelines. In response to a new VOC causing high morbidity and mortality, a quick evaluation of the half-lives of NAb to the new VOC by using longitudinal serum samples from clinical trials or research programs of different primary-series vaccinations and/or one or two boosters, together with measurement of existing NAb, could provide an important framework to determine the optimal time of a booster dose in each individual rather than recommendations for universal boosting. The primary-series vaccination subgroups include fully vaccinated COVID-19-naive participants with or without breakthrough infection and fully vaccinated COVID-19-recovered participants with or without breakthrough infection, following one or two booster doses of mRNA vaccine, including the new bivalent vaccine or other vaccine regimens.

Comparing the NT_50_ titers against different VOC over time, we found some individuals with little or no neutralizing activity at 3 and 7 months postvaccination with the Pfizer vaccine (Fig. 2G); all of these were healthy adults (22 to 74 years old) without immunocompromised conditions. In the analysis of sequential samples, higher NT_50_ titers at later time points were observed in three Moderna and four Pfizer vaccinees (Fig. 3B to C, Fig. 3E and G). None of these had more than a 4-fold increase in the NT_50_ titers at the later time point, which was in agreement with the lack of seroconversion of anti-nucleocapsid antibody in ELISA and ruled out the possibility of breakthrough infections between time points. Four vaccinees had a slight increase between the second and the third time points, probably reflecting fluctuating NT_50_ titers with a trend of slow decline from the first to the third time points. The other three had an increase between the first and second time points, probably reflecting the delayed appearance of peak NT_50_ titers after vaccination as reported previously (14, 15). When we compared vaccinees with the NI group, we found that the NI group had a greater fold reduction in NT_50_ titers against the omicron and less reduction against the delta variant (Fig. 1B and C), raising the possibility of infection by the delta variant. The infection dates of the NI group were between August and September 2020, suggesting that they were infected by the B.1 or D614G variant rather than the delta variant. It is worth noting that previous studies including systematic analysis and pooled analysis revealed a range of fold reduction in NT_50_ titers against the delta variant with individual heterogeneity among the NI group (18–20, 61–63). Consistent with previous reports that a large reduction in neutralizing activity against the omicron variant in the NI group, presented as NT_50_ titers below the limit of detection, was observed in the majority of cases (64% to 96%), most (66%) of our NI group had NT_50_ titers against the omicron variant that were below the limit of detection (21, 22).

We also compared 50% plaque reduction neutralization test (PRNT_50_) titers against the USA-WA1 strain, which contains the same S protein sequence (D at residue 614) as the mRNA vaccines, and NT_50_ titers against each VOC (Fig. S7). We found that the PRNT_50_ titers to live SARS-CoV-2 USA-WA1 strain (D614) correlated significantly with the pseudovirus NT_50_ titers against VOC, with a stronger correlation to the D614G strain and alpha variant than to the gamma and delta variants, supporting the notion that cross-neutralization induced by the S protein (D614) immunogen in the monovalent mRNA vaccines contributes to the neutralizing activities against different VOC. As new VOC continue to emerge, different strategies, such as bivalent or multivalent S protein-based vaccines or heterologous immunization with S proteins derived from the original strain (D614) and different VOC, have been proposed and/or generated to increase the breadth and potency of the induced NAb (59, 64).

There are several limitations to this study. First, although 87 samples were included from 51 COVID-19-naive participants at three time points, the sample size at each time point (*n* = 12 to 17 per group) was modest. Second, because the study participants were all healthy adults without immunocompromised conditions, the calculated half-lives of NT_50_ titers and the time for booster dose cannot be applied to other populations. Future studies involving larger sample of healthy adults, as well as different populations such as the elderly, adolescents, children, and immunocompromised hosts, are warranted to verify these observations and identify new parameters. Third, probably due to the small sample size, longer half-lives of NAb against VOC in the Moderna than in the Pfizer group were not significant based on sequential samples. Of note, the half-lives of NAb were significantly longer for the Moderna than for the Pfizer group based on the analysis of initial nonsequential samples. Future studies should include power analysis in their design.

In summary, we performed a detailed analysis of the kinetics and durability of NAb against different VOC following two doses of mRNA-based COVID-19 vaccines and found that the average time it took for NT_50_ titers to decline to 24, a reference level of protection, was longer in the Moderna group than in the Pfizer group. This finding could account for the slower decline in VE of the Moderna than the Pfizer vaccine observed in real-world settings and supports our hypothesis that measuring the NT_50_ titers against VOC together with information on NAb half-lives can determine the time at which NT_50_ titers decline to a reference protective level and provide a framework to determine the optimal time of a booster dose against VOC at the individual level. This is highly relevant in response to future VOC with high morbidity and mortality, as well as to other vaccines against pandemic viruses in the future.

## MATERIALS AND METHODS

### Human subjects.

The study was approved by the institutional review boards of the University of Hawaii (2020-00406) and Kaohsiung Medical University Hospital (KMUHIRB-E-I-20200013). With informed consent, coded plasma or serum samples were collected from participants in our COVID-19 cohort in Honolulu, Hawaii between July 2020 and October 2021 (41, 42), including 6 convalescent-phase samples from reverse transcription-PCR (RT-PCR)-confirmed COVID-19 cases (~3 weeks after a positive test), 12 postvaccination samples from COVID-19-recovered participants (~3 weeks following the second dose of Moderna or Pfizer vaccine, *n* = 6 in each group), and 87 postvaccination samples from 51 COVID-19-naive participants (~3 weeks, 3 and 7 months following the second dose of Moderna or Pfizer vaccine, *n* = 26 or 25, respectively), of whom 18 provided 3 sequential samples (*n* = 9 in each group) and 33 provided a single sample (*n* = 17 or 16 in each group) (Tables S1 and S3). In addition, 5 convalescent-phase samples were obtained from RT-PCR-confirmed COVID-19 hospitalized cases (~3 weeks after a positive test) at the Kaohsiung Medical University Hospital, Kaohsiung, Taiwan (Table S3). All participants were otherwise healthy adults without immunocompromised conditions based on a self-reported questionnaire. All samples from COVID-19-naive participants were tested by anti-nucleocapsid protein ELISA (Bio-Rad Platelia) to confirm no breakthrough infection during the study period.

### Plasmids.

Plasmids pNL4-3 R-E-miRFP, which contains the miRFP gene replacing the Luc gene of an *env*-defective HIV-1 reporter construct pNL4-3.Luc.R-E-, and D614G, which contains the S gene of the SARS-CoV-2 Wuhan-Hu-1 strain with D614G mutation and C-terminal 19-residue truncation, have been described previously (41). The S genes (alpha, beta and gamma VOC) were synthesized by two fragments (residues 1 to 461, flanked by KpnI and AflII [an introduced silent site mutation] sites; and residues 461 to 1,254, flanked by AflII and NotI sites), and cloned into the plasmid D614G (with KpnI and NotI sites) by 3-fragment ligation (Integrated DNA Technologies and Genewiz) to generate each plasmid. Two-step (residues 1 to 461 first, followed by residues 461 to 1,254) and one-step cloning of four fragments (residues 1 to 461, 461 to 853, and 853 to 1,254 by NEBuilder HiFi DNA assembly kit) (New England Biolabs) were performed to generate plasmids delta and omicron, respectively. All plasmids were confirmed by sequencing of the entire S gene insert (Fig. S1) and verified for expression by transfection and Western blot analysis (41, 43).

### SARS-CoV-2 pseudovirus.

To generate pseudoviruses for neutralization, HEK-293T cells were seeded into a 10-cm dish 1 day before transfection, co-transfected with 12 μg pNL4-3 R-E-miRFP and 3 μg of S plasmid using lipofectamine 2000, and incubated in Dulbecco’s modified Eagle medium (DMEM) containing 10% fetal bovine serum (FBS) (41). The supernatants were collected at 48 h posttransfection, followed by low-speed centrifugation at 300 × *g* for 10 min, then aliquoted and stored at −80°C. Previously, 1.65 × 10^9^ RNA copies of pseudovirus (~75 μL of D614G) per well, which resulted in miRFP signals 10 times higher than the mock-infected well at 72 h postinfection, were used for each neutralization test (41). To titrate each pseudovirus, supernatants were serially diluted 3-fold and used to infect HEK-293ThACE2 cells by spin infection (41); miRFP signals were quantitated at 72 h postinfection, and the amount of pseudovirus that resulted in miRFP signals 10 times higher than the mock-infected wells was used for the neutralization test.

### Pseudovirus neutralization test.

HEK-293T-hACE cells (2 × 10^4^ cells/well) were seeded onto 96-well plates 1 day prior to infection. Pseudovirus (D614G, alpha, beta, gamma, or delta) was mixed with 4-fold serial dilutions of serum at a 1:1 ratio, incubated at 37°C for 1 h, and added to each well for spin infection. At 72 h, the plates were scanned by a Li-Cor Odyssey imager (41). The percentages of infection at different serum dilutions (1:10 to 10,240) were calculated by the formula (intensity of serum + pseudovirus – intensity of medium only)/(intensity of pseudovirus only – intensity of medium only) × 100. Neutralization was calculated by the following formula: %neutralization = 100 – %infection (41). The NT_50_ titer was defined as the serum dilution that reached 50% neutralization using a 4-parameter nonlinear regression analysis (GraphPad v6.0) (Fig. S8) (41). A NT_50_ titer of <10 was arbitrarily assigned a value of 5.

### PRNT.

This assay was performed as previously described (41, 42). Briefly, Vero E6 cells (ATCC CRL-1586) had been grown in 6-well plates seeded at 2 × 10^5^ cells/well in 1× DMEM (2% HEPES, 1% penicillin-streptomycin) with 10% FBS 3 days prior and incubated at 37°C to achieve 100% confluence. Each serum sample was serially diluted (1:10, 1:40, further serial 2-fold) in 1× DMEM with 2% FBS and incubated with an equal volume of 50 to 100 PFU of SARS-CoV-2 isolate USA-WA1 (BEI Resources) at 37°C for 30 min, followed by inoculation into Vero E6 cells at 37°C for 1 h and overlay with 2% agar (2× DMEM with 4% FBS). Two days postinfection, a second overlay containing 0.33% Neutral red was added and plaques were recorded 12 to 24 h later. Sigmoidal dose-response with a variable slope simple logistical regression model was used to determine titers at 50% (PRNT_50_) neutralization (GraphPad v9.0).

### Decay rate and half-life analysis.

NT_50_ titer data were log-transformed and analyzed by VOC and vaccine type based on LME models, which estimated the NT_50_ titer decline while incorporating the random effects due to correlations among values from the same individual. The half-life of NAb against each VOC for each vaccine was determined based on the fixed effect. To compare the half-lives of NAb against each VOC between the two vaccines, the half-life in each participant was first calculated using the respective fixed effect estimate, and then compared by a Wilcoxon rank-sum test. Statistical modeling and tests of the NT_50_ titer data were performed using the *lme4* package in R version 4.1.2. Based on the half-life of NAb against each VOC and the NT_50_ titer on the sampling day, the time it took for NT_50_ titers to decline to 24 following the second-dose vaccine was calculated by the following formula: time (days) = sampling day + log (NT_50_ titer/24)/log 2 × half-life of NAb against that VOC.

### Statistical analysis.

A two-tailed Fisher’s exact test was used to compare qualitative variables between two groups. The two-tailed Mann-Whitney test and Wilcoxon signed-rank test were used to compare quantitative variables between two groups and within groups, respectively (GraphPad v6.0). The two-tailed Spearman correlation test was used to determine the relationship between NT_50_ and PRNT_50_ titers (GraphPad v6.0).

### Data availability.

The data sets supporting the conclusions of this article are included within the article and supplemental material.
