# Supplementary material for: Determining the Time of Booster Dose Based on the Half-Life and Neutralization Titers against SARS-CoV-2 Variants of Concern in Fully Vaccinated Individuals
Source: Microbiol Spectr. 2023 Jul 10;11(4):e04081-22. doi: 10.1128/spectrum.04081-22 (PMC10434144; doi:10.1128/spectrum.04081-22)
Supplement: Supplemental file 1 — Supplemental material. Download spectrum.04081-22-s0001.pdf, PDF file, 0.5 MB [file spectrum.04081-22-s0001.pdf]

Table S1. Characteristics of the Moderna and Pfizer vaccine groups

Characteristics	Moderna group	Pfizer group	p-value
Fist time point (~3 weeks)	n=16	n=17	
Sampling days post-dose 2 (mean [range])	19.3 [16–26]	16.3 [12–22]	[a]p=0.02
Age (years, mean [range])	35.7 [23–70]	39.2 [21–69]	[a]p=0.94
Gender (male/female)	7/9	9/8	[b]p=0.73
Race/ethnicity (A/W/mixed/Hispanic)[c]	11/5/0/0	10/5/0/2	[c]p=1
Second time point (~3 months)	n=13	n=14	
Sampling days post-dose 2 (mean [range])	94.4 [74–136]	77.0 [42–124]	[a]p=0.16
Age (years, mean [range])	40.9 [20–76]	37.0 [20–69]	[a]p=0.34
Gender (male/female)	6/7	8/6	[b]p=0.71
Race/ethnicity (A/W/mixed/Hispanic)[c]	10/3/0/0	9/5/0/4	[c]p=0.68
Third time point (~7 months)	n=15	n=12	
Sampling days post-dose 2 (mean [range])	215.7 [173–250]	217.6 [184–247]	[a]p=0.74
Age (years, mean [range])	40.4 [23–68]	39.2 [20–69]	[a]p=0.42
Gender (male/female)	9/6	8/4	[b]p=1
Race/ethnicity (A/W/mixed/Hispanic)[c]	9/5/1/0	7/5/0/2	[c]p=1
Sequential samples (from ~3 weeks to 7 months)	n=9	n=9	
Age (years, mean [range])	37.0 [23–68]	36.9 [21–74]	[a]p=0.65
Gender (male/female)	4/5	6/3	[b]p=0.64
Race/ethnicity (A/W/mixed/Hispanic)[c]	7/2/0/0	5/4/2/0	[c]p=0.62

[a] Mann-Whitney U test, two-tailed.
[b] Fisher's exact test, two-tailed.
[c] A: Asian, W: White, Fisher's exact test, two-tailed, comparing Asian and White.

Table S2. Days after the second dose of mRNA vaccine when NT_{50} titers decline to 24

Moderna group						Pfizer group					
**ID**	**Sampling day**	**Days NT_{50} titer=24[a]**				**ID**	**Sampling day**	**Days NT_{50} titer=24[a]**			
		**D614G**	**alpha**	**beta**	**delta**			**D614G**	**alpha**	**beta**	**delta**
First sequential samples						First sequential samples					
KMC08	16	390	470	256	368	VX05	13	357	291	186	293
VX21	16	251	287	186	230	VX19	13	240	286	170	241
VX22	24	243	303	156	233	VX56	14	239	337	248	245
VX29	16	316	332	342	266	VX59	13	220	315	253	178
VX53	26	225	276	87	255	VX73	14	219	176	56	318
VX30	19	246	313	98	242	VX72	14	216	265	276	165
VX69	15	286	320	174	371	VX04	12	376	373	209	321
VX134	19	358	353	189	255	VX09	95	194	356	185	207
VX136	19	483	446	207	253	VX74	14	232	245	26	100
Single time-point samples						Single time-point samples					
KMC22	19	373	320	331	253	VX38	22	361	318	211	273
VX23	19	428	436	369	314	VX40	22	227	191	103	237
VX28	20	432	429	347	329	VX42	17	263	251	177	234
VX51	20	392	348	314	253	VX44	19	354	277	208	314
VX61	21	394	355	296	273	VX50	21	268	191	151	210
VX62	21	335	303	280	240	VX85	21	239	182	93	203
VX16	18	255	327	125	259	VX35	14	214	223	110	251
VX31	82	415	507	231	400	VX82	19	176	160	64	128
VX104	87	140	93	−	148	VX75	15	222	62	235	290
VX70	74	281	251	150	160	VX07	88	134	269	−	139
VX97	124	180	143	−	−	VX88	82	−	147	−	120
VX24	206	311	287	−	271	VX108	76	268	268	246	246
VX71	221	−	317	−	−	VX112	118	164	175	126	122
VX81	207	448	−	314	410	VX126	124	273	266	179	212
VX128	213	356	294	−	249	VX79	202	227	−	−	−
KMC33	222	298	263	279	258	VX95	184	382	414	326	368
VX105	173	285	324	205	274						
Average		325	324	235	274	Average		253	252	174	226
Range		140-483	93-507	87-369	148-410	Range		134-382	62-414	26-326	100-368
Second and third time-point sequential samples						Second and third time-point sequential samples					
KMC08	136	273	397	285	181	VX05	51	262	248	184	231
	208	331	345	257	230		203	294	304	−	225
VX21	83	239	298	83	195	VX19	97	154	241	−	126
	234	332	334	351	266		245	−	−	−	−
VX22	108	320	393	−	345	VX56	46	231	279	199	233
	227	339	447	−	357		219	357	357	−	317
VX29	86	331	361	253	300	VX59	107	149	235	−	153
	227	337	367	313	299		198	244	224	−	213
VX53	58	209	243	85	192	VX73	42	169	102	61	191
	211	221	217	−	211		247	−	318	281	−
VX30	86	207	247	92	190	VX72	42	216	226	134	102
	212	324	355	−	257		247	299	337	−	−
VX69	131	164	191	−	272	VX04	68	371	345	190	349
	250	−	−	−	−		212	414	402	252	348
VX134	86	359	325	175	155	VX09	195	−	433	229	293
	212	367	438	281	281		244	261	249	266	321
VX136	86	323	399	165	147	VX74	42	221	277	−	119
	212	362	373	−	261		247	−	−	−	−

[a] Based on the half-life and NT_{50} titer against VOC on sampling days, the time after the second dose when NT_{50} titer declined to 24 was calculated. −, not determined due to NT_{50} titer <24.

Table S3. Characteristics of COVID-19-recovered plus vaccine and nature infection groups

**Characteristics**[a]	**NI +Moderna or Pfizer group**	**NI group**	**p-value**
Sampling time point (~3 weeks)	n=12	n=11	
Sampling days post-dose 2 or NI (mean [range])	20.2 [16–26]	25.3 [16–41]	[b]p=0.84
Age (years, mean [range])	49.8 [27–70]	48.2 [24–67]	[b]p=0.16
Gender (male/female)	5/7	7/4	[c]p=0.41
Race/ethnicity (A/W/mixed/Hispanic)[d]	7/3/2/0	6/4/1/0	[c]p=1

[d] NI, natural infection.
[b] Mann-Whitney U test, two-tailed.
[c] Fisher's exact test, two-tailed.
[d] A: Asian, W: White, Fisher's exact test, two-tailed, comparing Asian and White.

FS1

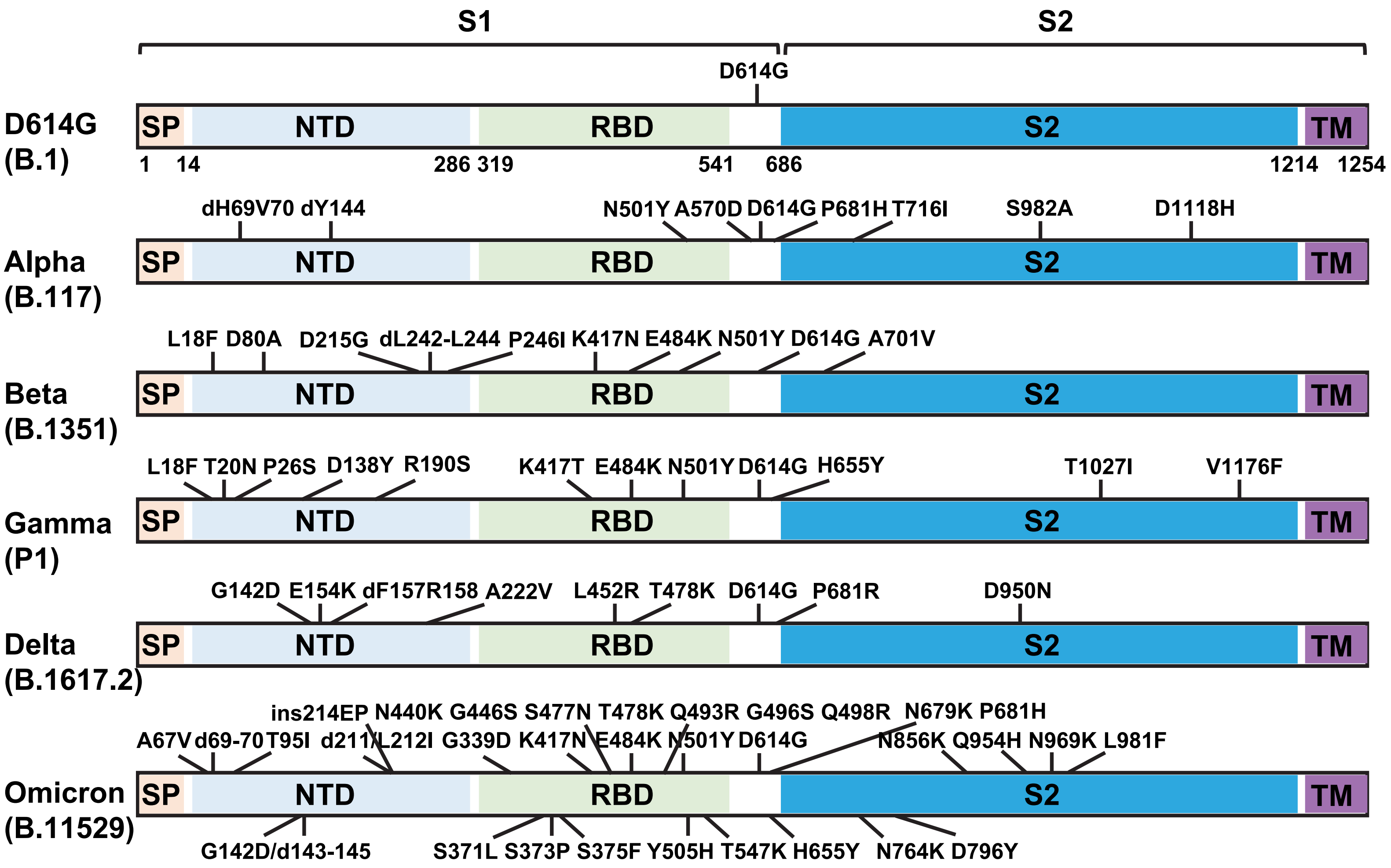

FIG S1 Plasmids expressing the S proteins of VOC and their mutations generated in this study. SP, signal peptide; S1 and S2, S1 and S2 subunits of the S protein; NTD, N-terminal domain; RBD, receptor binding domain; TM, transmembrane domain (43).

FS2

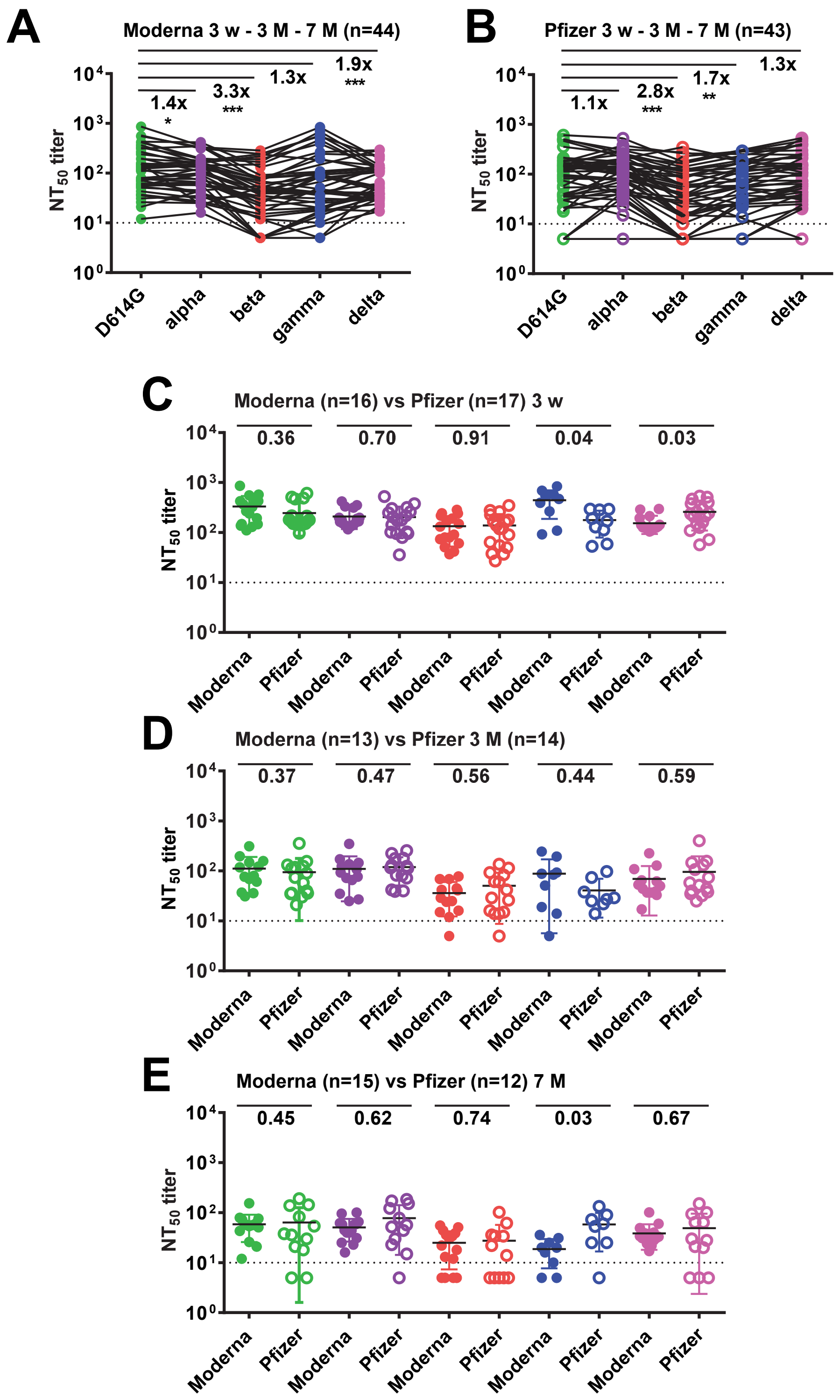

FIG S2 Comparison of NT_{50} titers against SARS-CoV-2 D614G strain and VOC (alpha, beta, gamma and delta) between COVID-19-naïve participants receiving Moderna and Pfizer vaccine at three time points. NT_{50} titers were based on pseudovirus neutralization test. (A,B) All samples together (n=44 and n=43 for the Moderna and Pfizer groups, respectively). (C) 3 weeks, (D) 3 months, and (E) 7 months. Number=p-value, the two-tailed Mann-Whitney test. Data are the means of duplicates from one experiment. Dot lines, NT_{50} titer=10. Color code for each VOC: green, D614G; purple, alpha; red, beta; blue, gamma; pink, delta.

FS3

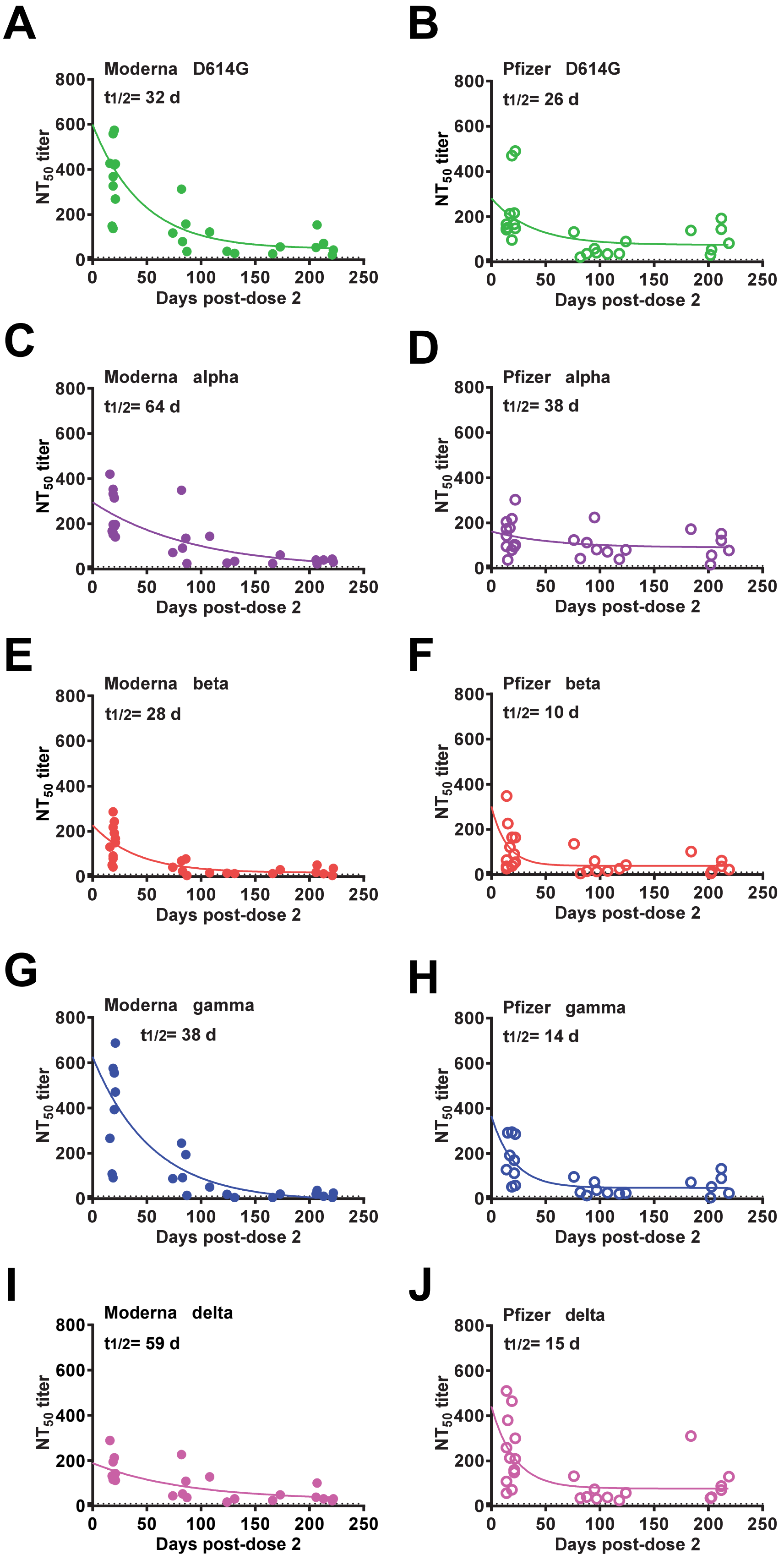

FIG S3 Decline of NT_{50} titers against VOC in the Modena and Pfizer groups. For the initial samples of the Moderna (A,C,E,G, n=26) and Pfizer (B,D,F,H, n=25), NT_{50} titers against the D614G strain (A,B) and each VOC including the alpha (C,D), beta (E,F), gamma (G,H) and delta (I,J) variants were plotted against sampling days for each individual following the second dose of the Moderna and Pfizer vaccine. A non-linear regression method (one-phase decay, least squares) was used to determine the best fit curve and estimate the half-life (GraphPad 6.0). Data are the means of duplicates from one experiment. Dot lines, NT_{50} titer=10.

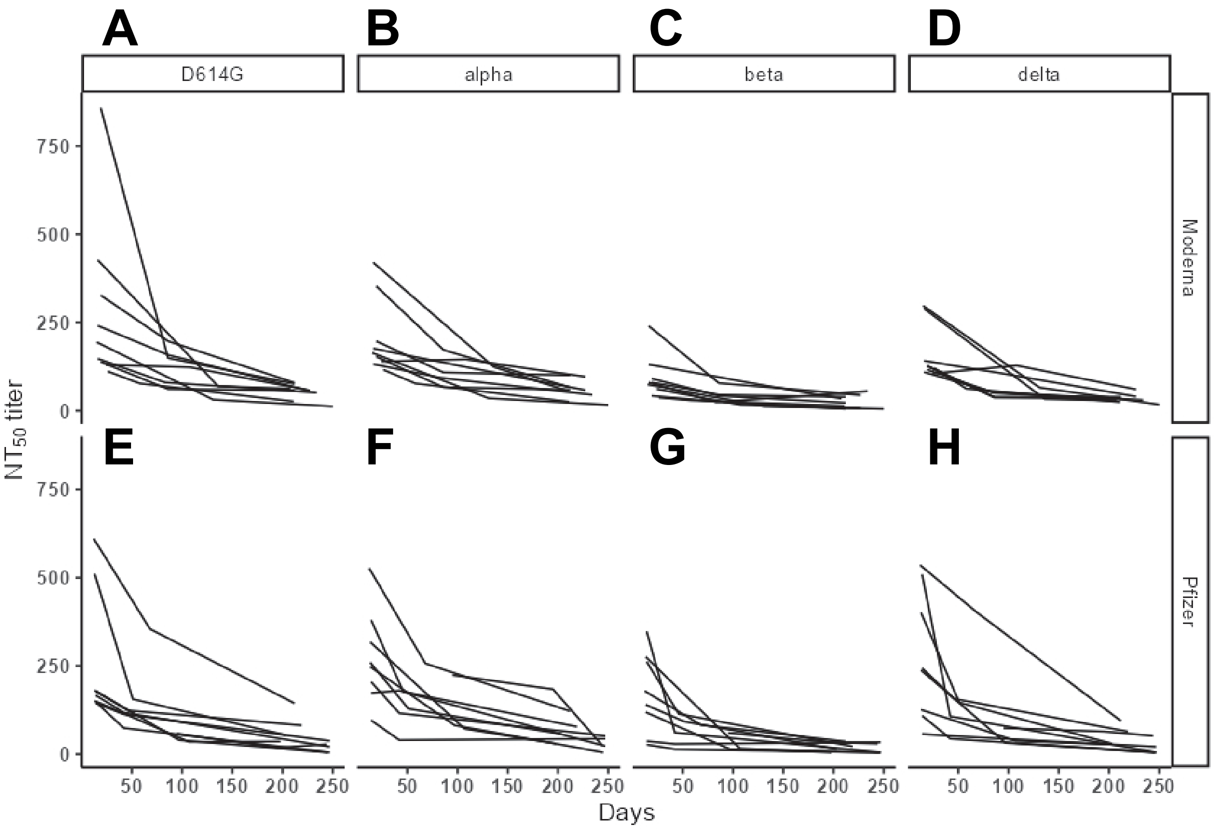

FIG S4 Decline of NT_{50} titers against VOC in the Modena and Pfizer groups. For the sequential samples of the Moderna (A−D) and Pfizer (E−H) groups (n=27 from 9 participants in each group), NT_{50} titers (before log-transformation) against different VOC were plotted against sampling days for each individual following the second dose of the Moderna and Pfizer vaccine. Data are the means of duplicates from one experiment.

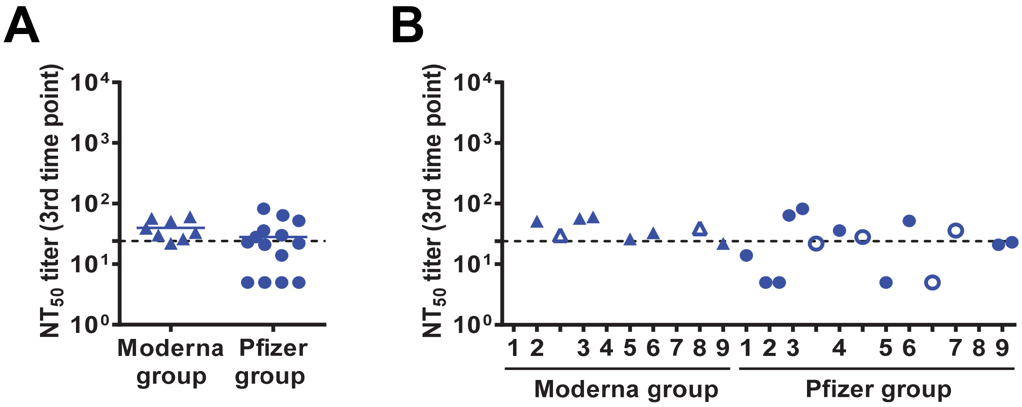

FIG S5 Comparison of NT_{50} titers of the third time point, which were within 30 days of the projected times based on the first time point, to 24. (A,B) Available data from the Moderna and Pfizer vaccinees as a group (A) or from each individual (B). NT_{50} titers against either D614G strain, alpha, beta or delta VOC were included in the analysis. Data are the means of duplicates from one experiment. Dash lines indicate NT_{50} titer=24. For panel B, NT_{50} titers higher or lower than 24 when the third time points were before or after the projected times, respectively, are presented as close symbol; otherwise open symbols. Numbers indicate each individual.

A

Moderna group						Pfizer group					
ID	Sampling day	Days NT_{50} titer=10				ID	Sampling day	Days NT_{50} titer=10			
		D614G	alpha	beta	delta			D614G	alpha	beta	delta
First sequential samples						First sequential samples					
KMC08	16	503	609	380	492	VX05	13	456	394	272	380
VX21	16	365	425	310	354	VX19	13	339	388	256	329
VX22	24	357	442	280	356	VX56	14	338	439	334	332
VX29	16	429	471	465	390	VX59	13	318	417	338	265
VX53	26	339	415	211	378	VX73	14	317	278	142	405
VX30	19	360	452	222	366	VX72	14	315	367	362	252
VX69	15	400	459	298	494	VX04	12	475	475	295	408
VX134	19	472	492	313	378	VX09	95	293	458	271	294
VX136	19	597	585	331	377	VX74	14	331	347	111	187
Single-time-point samples						Single-time-point samples					
KMC22	19	487	459	455	377	VX38	22	460	421	296	361
VX23	19	541	575	493	438	VX40	22	325	293	189	325
VX28	20	546	568	471	452	VX42	17	361	353	262	321
VX51	20	505	486	438	377	VX44	19	452	379	294	401
VX61	21	508	494	420	397	VX50	21	367	294	237	297
VX62	21	448	442	404	364	VX85	21	337	284	179	290
VX16	18	369	466	248	383	VX35	14	313	325	196	338
VX31	82	529	646	355	523	VX82	19	275	262	150	216
VX104	87	253	232	−	272	VX75	15	320	165	321	377
VX70	74	394	389	273	283	VX07	88	232	371	128	226
VX97	124	294	282	181	199	VX88	82	165	250	−	207
VX24	206	425	426	289	395	VX108	76	366	370	332	333
VX71	221	317	456	221	332	VX112	118	262	277	212	209
VX81	207	562	339	437	534	VX126	124	371	368	265	299
VX128	213	469	433	239	373	VX79	202	326	249	−	271
KMC33	222	411	402	403	382	VX95	184	480	516	412	455
VX105	173	399	463	328	398						
Average		434	458	339	387	Average		344	350	255	311
Range		253-597	232-646	211-493	199-534	Range		165-480	165-516	111-412	187-455

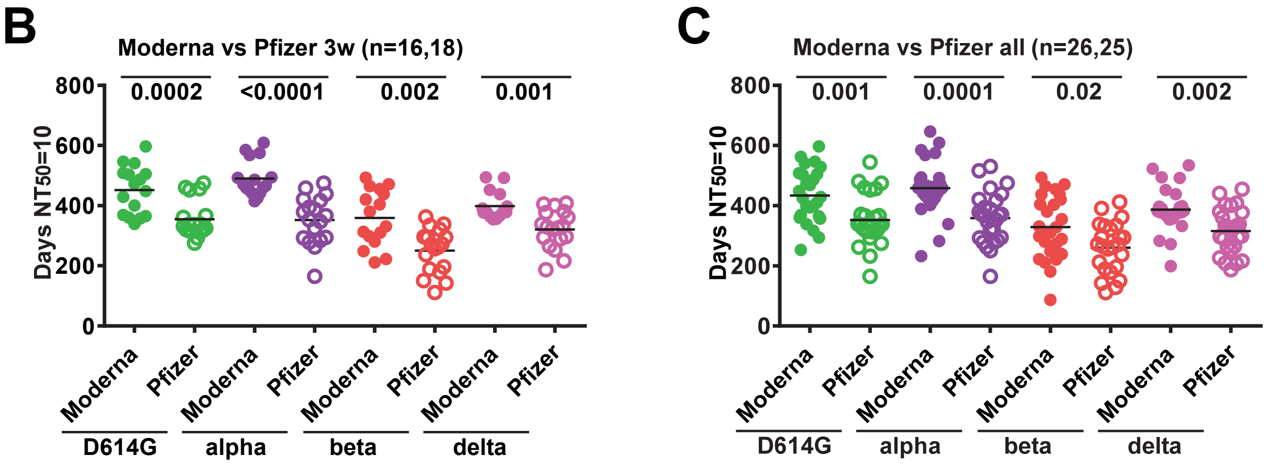

FIG S6 The time following the second dose of the Moderna or Pfizer vaccine when NT_{50} titers against each VOC declined to 10. (A) Based on the half-life and NT_{50} titer against each VOC on different sampling days (including the first sequential samples and single-time-point samples), the time after the second dose when NT_{50} titer declined to 10 was calculated for each participant in the Moderna and Pfizer groups. −, not determined due to NT_{50} titer <10. (B,C) Comparison of the time when NT_{50} titers declined to 10 for each VOC between the Moderna and Pfizer groups based on the 3 week samples (B) and all initial samples (C). Number=p-value, the two-tailed Mann-Whitney U test.

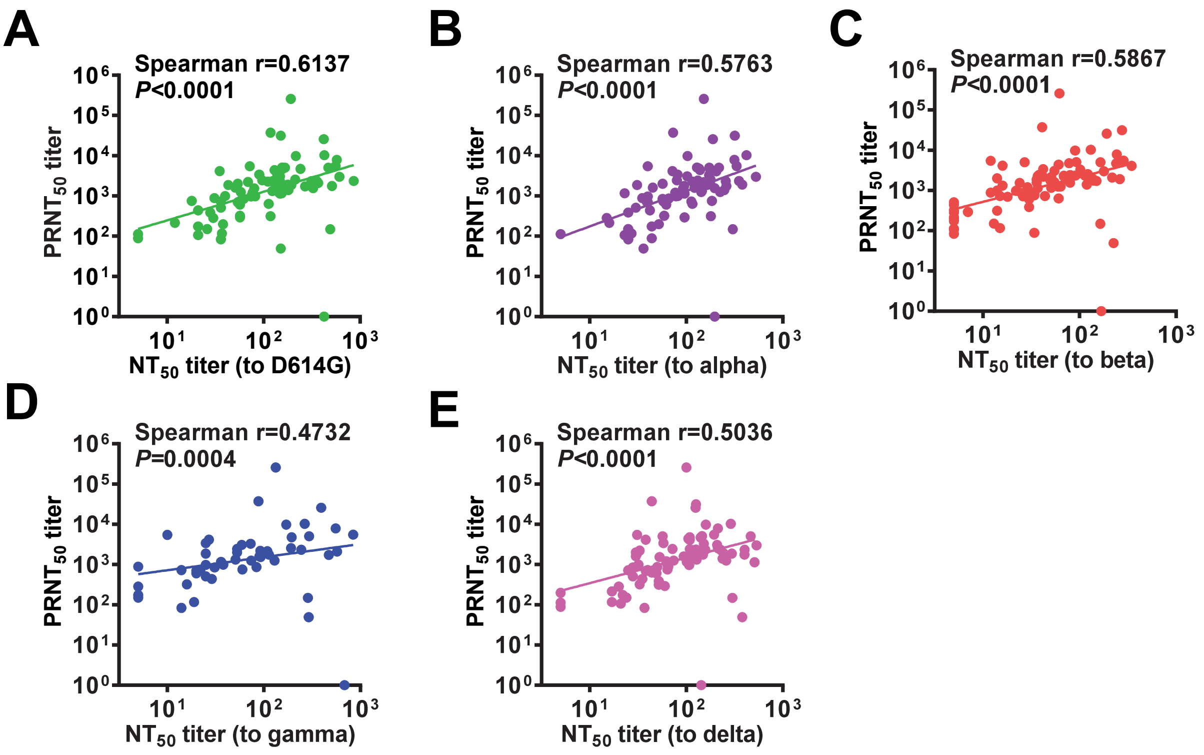

FIG S7 Correlation between $PRNT_{50}$ titers and NT_{50} titers against different VOC. (A−E) Relationship between $PRNT_{50}$ titers to USA-WA1 strain (containing D614) and pseudovirus NT_{50} titers against D614G strain (A), alpha (B), beta (C), gamma (D) or delta (E) variant. Data are the means of duplicates from one experiment. The two-tailed Spearman correlation test (GraphPad 6.0).

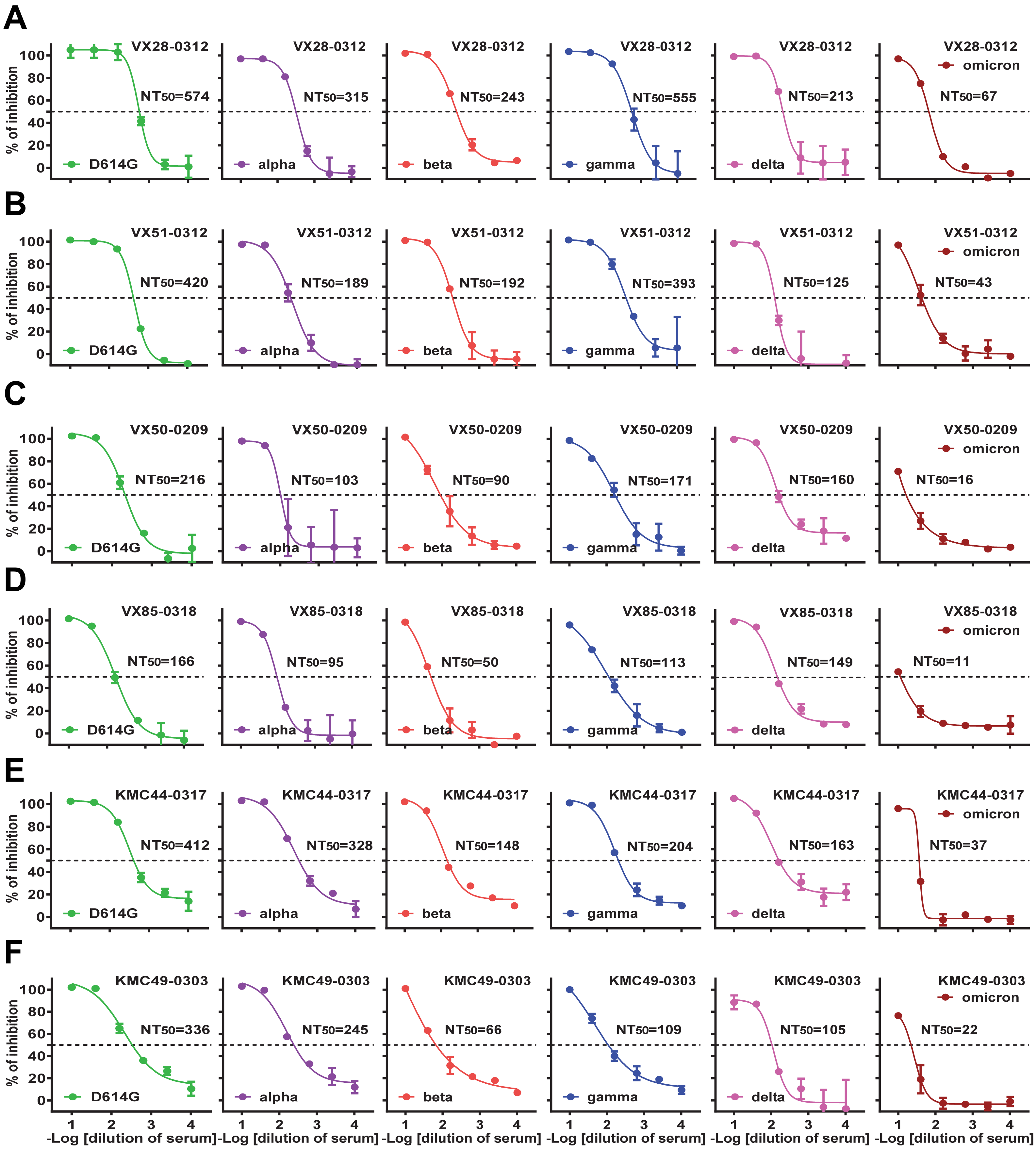

FIG S8 Neutralization curves and NT_{50} titers against different VOC in vaccinees by SARS-CoV-2 pseudovirus neutralization test. (A−F) Vaccinees including COVID-19-naïve participants after two doses of Moderna (A,B) or Pfizer (C,D) vaccine and COVID-19 recovered participants after two doses of Moderna vaccine (E,F) vaccine. Data are the means standard deviations of duplicates from one experiment.
